# The autonomic response following taVNS predicts changes in level of consciousness in DoC patients

**DOI:** 10.1038/s41598-024-84029-4

**Published:** 2025-03-01

**Authors:** Yan Li, Francesco Riganello, Jing Yu, Martina Vatrano, Mingquan Shen, Lijuan Cheng, Xiaohua Hu, Chengcheng Ni, Feiyang Wang, Bo Zheng, ChengCheng Zhang, Chaoyi Xie, Meiqi Li, Wangshan Huang, Fangfang Shou, Nantu Hu, Steven Laureys, Haibo Di

**Affiliations:** 1 International Vegetative State and Consciousness Science Institute, Hangzhou Normal University, Hangzhou, Zhejiang China; 2 School of Basic Medicine, Hangzhou Normal University, Hangzhou, China; 3 S. Anna Institute, Via Siris 11, Crotone, 88900 Italy; 4 Department of Rehabilitation, Hospital of Zhejiang Provincial Armed Police Crops, Hangzhou, China; 5 Coma Science Group, GIGA-Consciousness, University of Liège, Liège, Belgium; 6 Centre du Cerveau, University Hospital of Liège, Liège, Belgium; 7 Joint International Research Unit on Consciousness, CERVO Brain Research Centre, Laval University, Québec, Canada

**Keywords:** Disorders of consciousness, Transcutaneous auricular vagus nerve stimulation, Heart rate variability, Prognosis, Support vector machine, Diseases of the nervous system, Neurology

## Abstract

Advancements in emergency medicine and critical care have significantly improved survival rates for patients with severe acquired brain injuries(sABI), subsequently increasing the prevalence of disorders of consciousness (DoC) such as Unresponsive Wakefulness Syndrome (UWS) and Minimally Conscious State (MCS). However, the assessment of conscious states relies on the observation of behavioral responses, the interpretation of which may vary from evaluator to evaluator, as well as the high rate of misdiagnosis, which together pose significant challenges for clinical diagnosis. The study investigates the utility of transcutaneous auricular vagus nerve stimulation (taVNS) in modulating autonomic responses, as evidenced through heart rate variability (HRV), for distinguishing between healthy individuals and DoC patients and for prognosticating patient outcomes. A prospective randomized clinical trial was conducted from Februry 9, 2022, to February 4, 2024, at Hangzhou Armed Police Hospital in China. Healthy controls (HC) and DoC patients were enrolled in this study. The taVNS was administered to each subject for ten minutes. There electrocardiogram (ECG) signals were recorded for the analysis of HRV both during the stimulation and the ten minutes of rest that preceded and followed the stimulation. Subsequent investigations utilized Support Vector Machine (SVM) modeling, enhanced by a Radial Basis Function (RBF) kernel, to explore potential predictors of patient outcomes. This approach aimed to differentiate HC from DoC and MCS from UWS patients. 26 HC and 36 patients diagnosed with DoC were included in the analysis,. The DoC group consisted of 17 patients with a diagnosis of MCS and 19 with diagnosis of UWS/VS. Significant modulations in HRV parameters (HF, VLF, SampEn) were observed, indicating variations in autonomic response between the control group and DoC patients. Using the VLF, LF, and SampEn features in SVM model, DoC and HC were correctly classified with an accuracy of 86%. Similarly, MCS and UWS were classified with an accuracy of 78%. The SVM modeling achieved an 86% accuracy rate in predicting outcomes three months post-intervention, with a 71% confirmation rate at six months.The results highlight taVNS’s potential as a therapeutic modality in managing DoC by demonstrating its impact on autonomic regulation and suggesting pathways for enhancing recovery, which accentuates the significance of exploring brain-heart dynamics in DoC, presenting a novel approach to therapeutic strategies. **Trial Registration Information**: URL: chictr.org.cn; Unique identifier: ChiCTR2100045161. Date of the first registration: 9th/ April/ 2021.

## Introduction

Advancements in emergency medicine and critical care have notably increased survival rates for patients with severe acquired brain injuries (sABI) stemming from both traumatic and non-traumatic origins^1,2^. However, a proportion of these survivors may develop disorders of consciousness (DoC), manifesting as varying levels of awareness and arousal impairment^3^. DoC encompasses conditions like Unresponsive Wakefulness Syndrome (UWS), previously known as Vegetative State (VS), and Minimally Conscious State (MCS), which are further divided into MCS + and MCS-^4,5^. These states, characterized by differing levels of responsiveness and consciousness, present significant clinical diagnosis and management challenges, often leading to misdiagnoses. While the UWS display reflexive behavior but lack signs of consciousness^3,6^, the MCS patients demonstrate improved arousal levels and the ability to interact with the environment, such as visual tracking, object localization, or following commands^5,7^. The shifts between these conditions are dynamic with individual variability, yet they can be consistently observed and replicated^8^.

The diversity and subjective nature of consciousness in DoC patients pose significant challenges to accurate clinical diagnosis^9^, contributing to a misdiagnosis rate today of approximately 40%^10,11^.

Monitoring Autonomic Nervous System (ANS) activity provides essential insights into the physiological state of these patients, which might not be evident through behavioral assessments alone. Given that the ANS regulates crucial bodily functions such as heart rate (HR), blood pressure, and thermoregulation, its monitoring can be instrumental in assessing a patient’s level of arousal and responsiveness^12,13^. This is particularly significant in the challenge represented to differentiating the UWS/VS and MCS^14^as well as MCS + and MCS-^15,16^. Moreover, the assessment of the ANS can reveal signs of consciousness and cognitive functions that might not be apparent through conventional behavioral assessments, enhancing the accuracy of the prognosis^13,14,17^.

**Fig. 1:**
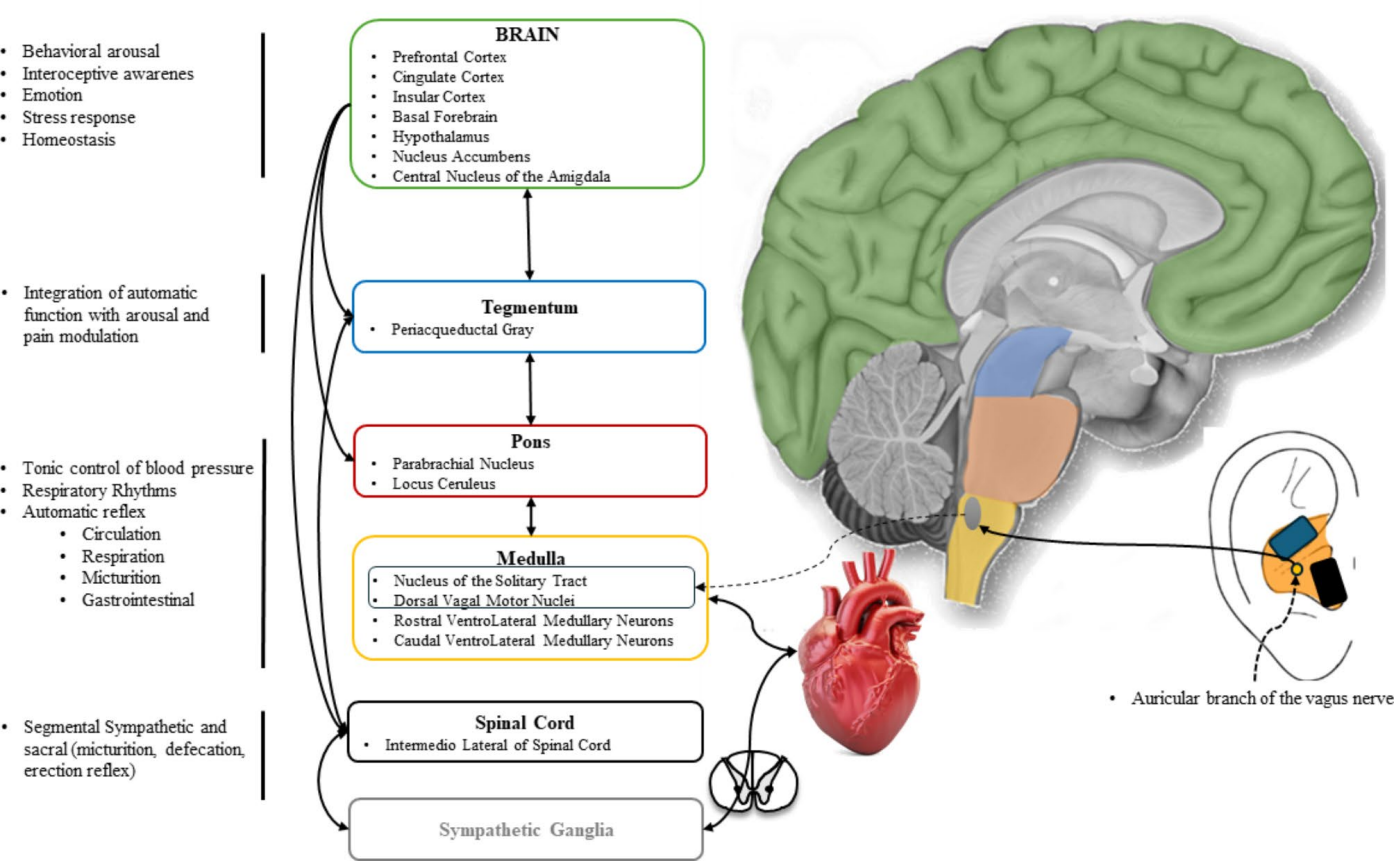
Schematic representation of the Central Autonomic Network and link with the auricular branch of the vagus nerve into the ear.

The Central Autonomic Network (CAN)^18^ model explains the ANS-CNS (i.e. central nervous system) interaction in homeostasis and response to stimuli, highlighting key brain regions involved in autonomic response generation (Fig. 1). This model emphasizes the interconnected roles of key forebrain regions (anterior cingulate, nucleus accumbens, insula, ventromedial prefrontal cortex, amygdala, hypothalamus) and the brainstem (periaqueductal gray, parabrachial nucleus, nucleus of the solitary tract, ventrolateral medulla). These central nervous system(CNS) structures, crucial in autonomic response generation, receive various sensory inputs and project to sympathetic and parasympathetic neurons^18–20^. The model also underscores the forebrain and brainstem’s roles in modulating autonomic outputs in response to pain, emotions, behavior, and cognitive processes. This comprehensive framework details the importance of the ANS-CNS interaction in physiological balance and adaptability and in generating specific autonomic responses to stimuli mediated by their projections to preganglionic sympathetic and parasympathetic neurons^20^.

The brain directly regulates the heart via the sympathetic and parasympathetic divisions of the ANS, and Heart Rate Variability (HRV) is a reliable, non-invasive measure of ANS function and ANS-CNS interaction, reflecting autonomic regulation of the heart and indirectly mirroring higher brain functions^19^. HRV is valued for its strong signal-to-noise ratio, simplicity, and cost-effectiveness, making it a prominent biomarker in neurophysiological studies^21^.

HRV has emerged as a vital tool in assessing and prognosticating DoC in recent years, given its role in understanding complex ANS and CNS interactions. HRV analysis is conducted in time, frequency, and non-linear domains, with non-linear analysis proving particularly insightful for understanding complex ANS and ANS-CNS interactions^22^. In HRV analysis, the low frequency (LF) reflects sympathetic and parasympathetic modulation in the frequency domain, while the high frequency (HF) predominantly indicates vagal activity. The measure in the non-linear domain, such as Sample Entropy (SampEn), provides information on the complexity and irregularity of HR time series, offering insights into the heart’s adaptive mechanisms and brain-heart interaction^22^.

Vagus nerve (VN), a significant portion of the parasympathetic nervous system, plays an essential bidirectional role in maintaining the ANS-CNS connection between the body and the brain, especially homeostasis^23^.

The VN is a mixed nerve with roughly 80% afferent and 20% efferent axons, predominantly composed of sensory fibers. These fibers vary in conduction speed and size, ranging from the fastest and largest Aα fibers to the smallest and slowest unmyelinated C fibers, with Aβ, Aγ, Aδ, and B fibers falling in between^24^. The external ear, particularly the tragus, concha, and cymba concha, is rich in cutaneous afferent vagal nerve pathways, making it a focal point for vagus nerve stimulation(VNS)^25^. Specifically, the cymba conchae is the primary area for stimulating the VN, as the auricular branch of the vagus nerve (ABVN) is almost entirely distributed here^26^. The ABVN mainly consists of Aβ, Aδ, and C fibers, with a similar number of Aβ fibers on both sides of the ABVN but significantly fewer than in the cervical branch of the VN^27^. This unique distribution makes the auricle an ideal target for transcutaneous Auricular Vagus Nerve Stimulation (taVNS).

The taVNS has gained popularity in clinical use due to its ease of use^28,29^. It uses microcurrent to stimulate specific points on the auricle, targeting the ABVN.

Its mechanism of action involves the complex interaction between CNS and ANS. The detailed understanding of the VN’s role, especially through taVNS, provides the theoretical basis for the expected outcomes in HRV modulation among DoC patients.

In stimulating DoC, the taVNS modulates thalamocortical connectivity and neurotransmitter systems, which is crucial for promoting awareness and arousal. This method focuses on modulating key brain networks involved in consciousness through a bottom-up neural transmission pathway, which potentially enhances brain network connectivity, neurotransmitters^30^, neuronal excitability^31^, and neuroplasticity^32^.

**Fig. 2:**
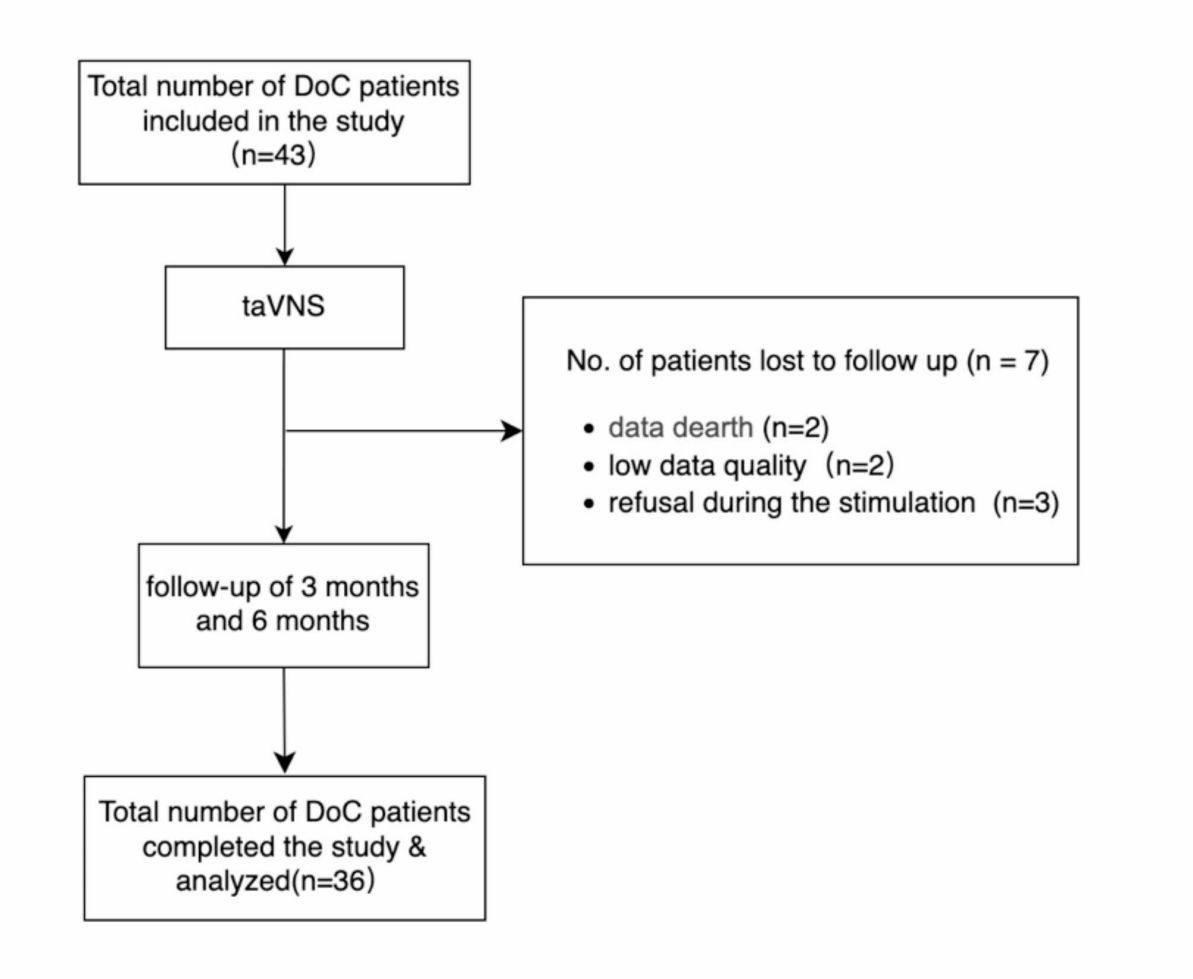
STROBE diagram.

Our work, conducted as an Event-Based single-session study, aims to harness these theoretical and anatomical insights into practical applications(Fig. 2). Specifically, we seek to: (i) identify differences in taVNS response, including autonomic and behavior responses, between healthy individuals and DoC patients and between UWS/VS and MCS subgroups by HRV as well as CRS-R, a standardized clinical assessment tool and (ii) explore the potential of taVNS and HRV analysis as prognostic tools in clinical settings. This approach will contribute to a more nuanced understanding of DoC and potentially offer new avenues for diagnosis and treatment.

## Methods

### Participant selection

The study included 26 healthy volunteers (HV; 8 males, mean age 51 ± 14 years; 18 females, mean age 49 ± 15 years) and 36 patients diagnosed with DoC (by completing five MCS assessments within one week). The DoC group consisted of 17 patients with a diagnosis of MCS (MCS; 16 males, mean age 62 ± 10 years, with etiologies of 6 traumatic, 9 hemorrhagic, and 1 infectious; 1 female, age 60, hemorrhagic) and 19 with diagnosis of UWS/VS (11 males, mean age 51 ± 21 years, with etiologies of 6 traumatic, 3 hemorrhagic, and 2 anoxic; 8 females, mean age 55 ± 11 years, with etiologies of 3 traumatic, 4 hemorrhagic, and 1 anoxic). The consciousness level of the patients was assessed using the coma recovery scale-revised (CRS-R)^3^.The mean duration since injury was 347 ± 334 days for the MCS and 291 ± 244 days for the UWS/VS subgroups (Table 1). The inclusion criteria were: (1) age 16 or older; (2) absence of neurostimulant drugs 48 h before the stimulation; (3) no neuromuscular function blocker administered within 24 h before enrollment; (4) no ear pain or previous hearing impairment; (5) no drugs that may affect heart rate and HRV parameters administered within 24 h before enrollment. The exclusion criteria were (1) personal or family history of seizures, cardiovascular disease, Parkinson’s disease, or a history of a progressive neurological or psychiatric disorder; (2) untreated cerebral edema; (3) injury, redness, or inflammation of the skin on the chest or wrist; (4) contusions, fractures or flaccid paralysis of the upper limbs; (5) mechanic ventilation; (6) history of neurological or psychiatric disorders; (7) history of paroxysmal sympathetic hyperactivity syndrome. All HC were instructed to avoid the consumption of alcohol, nicotine, or caffeine prior to the study to minimize any potential confounding effects on taVNS and HRV measurements.

**Table 1:** Demographic information and diagnosis.

Group	Etiology	Age	Gender	Time from injury	CRS-*R* total score	CRS-*R* subscores	Level of Consciousness	three months diagnosis	UO/FO three months prognosis	Six months diagnosis	Group	Age	Gender
Disorders of Consciousness	H	16–48	Male	880	9	2-1-2-2-0-2	UWS	dead	UO	dead	Healthy Control	24–35	Female
	T			98	7	1-0-2-2-0-2	UWS	UWS	UO	UWS			
	H			337	7	1-0-2-2-0-2	UWS	UWS	UO	MCS-			
	H			101	9	1-3-2-1-0-2	MCS-	UWS	UO	dead			
	H			399	10	1-3-2-2-0-2	MCS-	MCS+	FO	MCS+			
	H	52–59		50	6	1-0-2-1-0-2	UWS	dead	UO	dead			
	H			145	11	2-3-3-1-0-2	MCS-	MCS-	FO	MCS-		48–56	
	T			108	9	2-1-2-2-0-2	UWS	MCS-	FO	UWS			
	H			28	7	1-1-2-2-0-1	UWS	MCS+	FO	MCS+			
	H			600	12	2-3-2-2-1-2	MCS+	MCS+	FO	MCS+			
	H			56	18	3-4-5-2-1-3	MCS+	MCS+	FO	MCS+			
	H			125	13	1-4-5-1-0-2	MCS-	dead	UO	dead			
	T			700	13	4-1-3-2-1-2	MCS+	UWS	UO	MCS+			
	T	61–69		58	19	4-5-5-2-1-2	MCS+	UWS	UO	UWS		57–72	
	T			502	7	0-3-2-1-0-1	MCS-	MCS-	FO	UWS			
	T			1299	20	3-5-5-3-1-3	MCS+	EMCS	UO	EMCS			
	I			138	19	3-5-5-2-1-3	MCS+	EMCS	FO	MCS-			
	T			409	8	2-0-2-2-0-2	UWS	UWS	UO	UWS			
	T			116	11	1-3-4-1-0-2	MCS-	MCS-	FO	MCS-		24–53	Male
	T			239	8	2-1-2-1-0-2	UWS	UWS	UO	MCS-			
	T			435	9	2-1-2-2-0-2	UWS	MCS-	FO	UWS			
	H	70–77		69	10	1-3-2-2-0-2	MCS-	UWS	UO	MCS-			
	T			559	10	1-3-3-1-0-2	MCS-	UWS	UO	UWS		57–65	
	H			48	12	3-1-5-2-1-0	MCS+	MCS+	FO	MCS+			
	T			345	9	2-1-2-1-0-3	UWS	MCS+	FO	MCS+			
	H			554	8	1-1-2-2-0-2	UWS	dead	UO	dead			
	H			465	15	2-3-5-2-0-3	MCS+	EMCS	FO	EMCS			
	T	63–77	Female	406	7	0-1-2-2-0-2	UWS	dead	UO	dead	T: *traumatic* H: *hemorrhagic* I: *infection* UWS: *unresponsive wakefulness syndrome* MCS: *minimally conscious state* FO: *favorable outcome* UO: *unfavorable outcome*		
	H			511	13	2-5-2-1-1-2	MCS+	MCS-	UO	MCS-			
	H			99	7	2-0-2-1-0-2	UWS	MCS-	FO	MCS-			
	H			128	8	1-1-2-2-0-2	UWS	MCS-	FO	UWS			
	T	41–54		798	7	0-1-2-2-0-2	UWS	UWS	UO	UWS			
	H			106	5	0-0-2-2-0-1	UWS	UWS	UO	UWS			
	H			124	4	1-0-2-1-0-0	UWS	MCS-	FO	MCS-			
	T			229	8	1-1-2-2-0-2	UWS	MCS-	FO	UWS			
	H			161	6	0-0-2-2-0-2	UWS	UWS	UO	UWS			

### taVNS protocol

A prospective randomized clinical trial was conducted from Februry 9, 2022, to February 4, 2024, at Hangzhou Armed Police Hospital in China. Healthy controls (HC) and DoC patients were enrolled in this study (Table 1). Participants were positioned comfortably, either seated in a wheelchair or semi-reclined on a bed, within a controlled environment characterized by stable luminosity (100 lx) and temperature (20 ~ 24 °C) to minimize external variables.

TaVNS was administered for 10 min using the Ear Vagus Nerve Stimulator (model TENS-200 A, http://www.hwato-med.com/index.php/product/jjfaxq/21.html). The device’s electrodes were placed on the cymba conchae to effectively target the auricular branch of the VN. Stimulation parameters were set to emit a positive wave with a pulse width of 200 microseconds; the output frequency alternated between 4 Hz and 20 Hz in cycles of 3 and 7 s, respectively, to optimize neural engagement^33^.

Electrocardiogram (ECG) recordings were recorded, with a sampling rate of 250 Hz, using a three-lead monitoring device (Psychorus-Songshan, HuiXin, China, https://www.hxpsych.cn/bxsxdcjy), with adhesive electrodes positioned to the left clavicle (white), right clavicle (black), and left lower chest (red).

ECG data were collected during the 10-minute taVNS session and two resting states of 10 min: one preceding the stimulation (R1) and the other following it (R2). This approach was used to observe the autonomic changes elicited by taVNS, focusing on the comparative assessment between the stimulation phase (differences between taVNS and R1) and the restoration phase (differences between the second and the first resting state).

### Standard protocol approvals, registrations, and patient consents

The study protocol was approved by the Ethics Committee of Hangzhou Normal University (Approval No. 20190083) and all the participating centers. All participants or their legally authorized representatives provided written informed consent. The trial was registered on Clinicaltrials.gov (URL: chictr.org.cn; unique identifier: ChiCTR2100045161). All experiments were performed in accordance with the ethical standards of the Helsinki Declaration and applicable national regulations, as well as the STROBE statement to ensure a high standard of reporting that fosters transparency and thoroughness.

### HRV analysis

HRV analysis was conducted using Kubios HRV Premium software (version 3.1, https://www.kubios.com/hrv-premium/). The integrity of the acquired ECG signal was first verified, followed by identifying R-peaks employing the Kubios-specific QRS detection algorithm, which is grounded in the Pan-Tompkins method^34^. To accurately delineate R-peaks, cubic spline interpolation at 4 Hz was performed, complemented by a visual inspection to rectify any anomalies, including missing or ectopic beats, thus ensuring the accuracy of the HRV analysis. To address potential distortions caused by non-stationarity in the signal, a quadratic polynomial detrending method was applied to the RR interval time series. This procedure effectively minimized lower frequency influences on the power spectral density (PSD) calculations. The detrended RR interval sequence was then rigorously analyzed within both the frequency and non-linear domains to determine the HRV metrics.

PSD analysis was carried out over three distinct frequency bands: high-frequency (HF: 0.15–0.50 Hz), low-frequency (LF: 0.04–0.15 Hz), and very-low-frequency (VLF: 0.0033–0.04 Hz), employing the Fast Fourier Transform (FFT) technique with Welch’s method and 150-second window widths. Due to the skewed nature of spectral power distributions, a logarithmic transformation was performed to normalize the data, facilitating accurate statistical evaluation.

In the non-linear analysis, the SampEn was computed as the negative natural logarithm of the likelihood that two sets of simultaneous data points, within a set length m and tolerance r, remain similar at the next incremental point (m + 1). Parameters for the SampEn analysis — embedding dimension (m) and tolerance level (r) — were set at 2 and 0.2, respectively. The chosen tolerance level r was equivalent to 20% of the RR interval time series standard deviation.

### Support vector machine

This study employed the Support Vector Machine (SVM) technique to differentiate between favorable and unfavorable outcomes. SVMs effectively segregate distinct groups in a dataset by establishing an optimal boundary that maximizes their separation. This capability is particularly advantageous for complex datasets, as it balances the risk of overfitting (too closely fitting the data) against the need for a model that generalizes well to new data.

The SVM’s performance was enhanced using a Radial Basis Function (RBF) kernel. This kernel simplifies the input data, aiding in the distinction of otherwise indistinguishable data points. The synergy of SVM and the RBF kernel facilitates the management of complex data relations, leading to more accurate classifications.

Two parameters, cost (C) and RBF gamma, are pivotal in the SVM model. The C parameter ensures a balance between the margin maximization (distance from the decision boundary to the nearest data points) and the minimization of classification error. Meanwhile, the RBF gamma parameter influences the decision boundary’s shape, affecting the curve’s flexibility and smoothness. Appropriately adjusting these parameters is essential for balancing the model’s complexity and generalization ability, enhancing its performance on unseen data.

The model’s robustness and reliability were assured through ten-fold cross-validation, which divides the data into ten subsets for training and validation. This process involves repeatedly training and validating the model, each time with a different subset as the validation set and the remaining data for training. Such a method ensures that each data point is used for validation exactly once and for training in nine out of ten cases. The average performance across all validations provides a reliable measure of the model’s effectiveness, reducing overfitting risks and ensuring generalizability to new data. This approach also evaluates the model’s consistency across various training and validation scenarios, which is essential for confirming its reliability and practical applicability.

### Outcome

Given the study’s relatively modest cohort size and objectives, a unified binary classification system was adopted for all patients with MCS-, MCS+, and those transitioning from MCS. This binary stratification differentiates ‘favorable’ from ‘unfavorable’ outcomes, which is pivotal in identifying clear prognostic indicators amid the complex variability inherent in DoC.

A ‘favorable outcome’ has been defined to capture meaningful clinical changes: this includes any observable progression from unresponsive UWS/VS to any MCS classification or any maintenance or improvement in the CRS-R total score for patients already in an MCS state. This dichotomous categorization enhances the statistical power and interpretability of the SVM modeling outcomes, facilitating a more precise predictive analysis.

A follow-up assessment was scheduled for six months following the assessment to evaluate the long-term effects and changes in the level of consciousness, allowing for a comprehensive analysis of the patient’s recovery trajectories.

### Statistical analysis

The stimulation and restoring phases were considered in the analysis.

The two conditions were compared for the HRV spectral (natural logarithm of VLF, LF, and HF PSD, and SampEn) in HC, MCS, and UWS/VS groups by Wilcoxon exact test. DoC vs. HC and MCS vs. UWS/VS were compared using the Mann-Whitney exact test.

The effect size r was calculated as the absolute value of Z/√(N) for the Mann-Whitney test, and as absolute value of Z/√(2*N) for the Wilcoxon test, where Z is the Z-statistic of the statistical test, and N is the total number of subjects. The effect size results were considered: *r* < 0.1 not significant; 0.1 ≤ *r* < 0.3 low; 0.3 ≤ *r* < 0.5 medium; *r* > 0.5 high.

The analysis was considered two two-tailed tests with the level of significance *p* set to 0.05.

## Results

Comparing stimulation (VNS-R1) and restoring (R2-R1) conditions, at Wilcoxon exact test, a significant difference was found in UWS/VS for VLF (Z=-2.556, *p* = 0.008, *r* = 0.4) and SampEn (Z=-2.243, *p* = 0.024, *r* = 0.3) and in HC for HF (Z=-2.083, *p* = 0.037, *r* = 0.3).

**Fig. 3:**
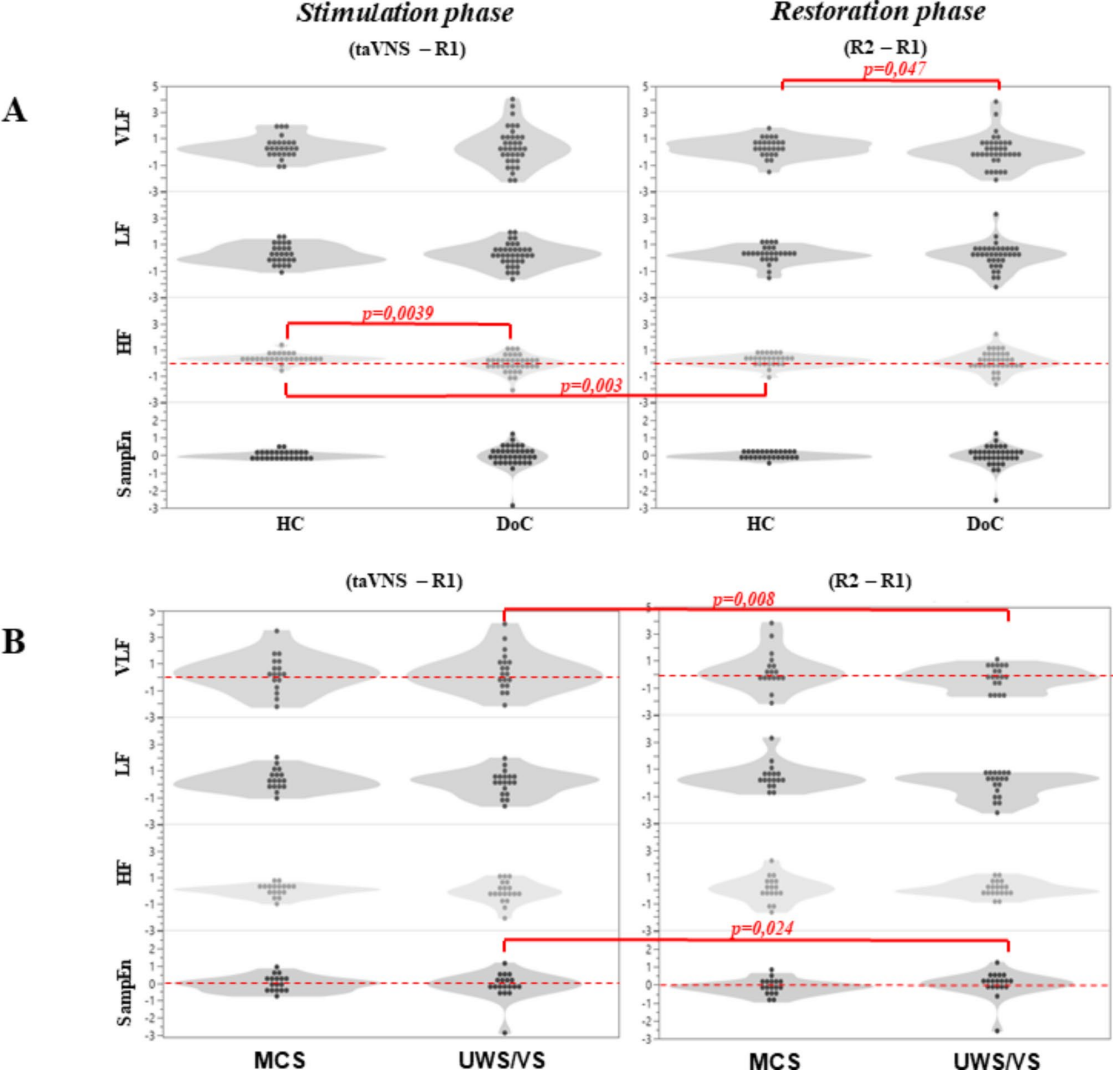
Violine plots. (**A**) violin plots relative to HRV parameters natural logarithm of the PSD of VLF, LF, HF, and SampEn of HC and DoC groups in taVNS-R1 and R2-R1 conditions. (**B**) violin plots relative to HRV parameters natural logarithm of the PSD of VLF, LF, HF, and SampEn of MCS and UWS/VS groups in taVNS-R1 and R2-R1 conditions. The red line indicates the significant differences between groups or conditions. The dashed red line is the zero to observe the significant differences better.

Comparing DoC and HC, at Mann-Whitney exact test, significant differences were found for VLF_R2−R1_ (Z=-1.990, *p* = 0.047, *r* = 0.3) and HF_VNS−R1_ (Z=-2.889, *p* = 0.003, *r* = 0.4). We observed significant changes in HF, VLF, and SampEn during VNS-R1 and R2-R1 conditions among HC and DoC patients. Notably, HC exhibited elevated HF during stimulation, while DoC patients showed higher VLF during recovery, while no significant differences were found comparing MCS and UWS/VS (Fig. 3).

### SVM performance

The input variables used in the SVM model were age, time from injury, etiology, and HRV parameters (i.e., the natural logarithm of the PSD of VLF, LF, and HF, and the SampEn). The stimulation (taVNS-R1) and restoring (R2-R1) conditions were considered separately. After feature selection, the selected input parameters for predicting UO and FO outcomes were VLF, LF, and SampEn.

C and gamma parameters were tested for each condition by comparing 100 different models, with C and gamma ranging between 0.5 and 300 and 0.01 and 1, respectively.

**Fig. 4:**
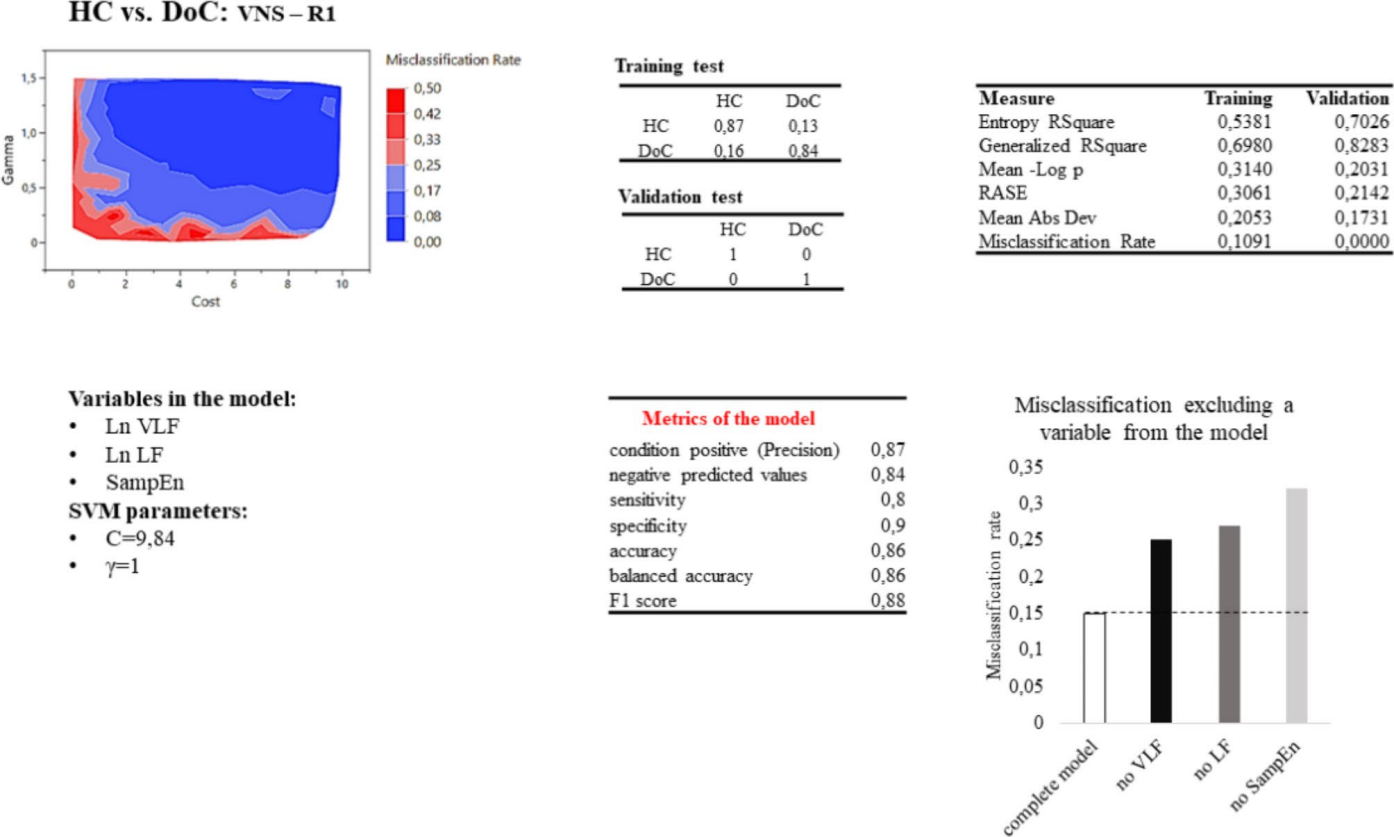
SVM HC/DoC classification results. Line above: misclassification rate of 100 SVM models based on cost and gamma values; confusion matrix of the SVM model in predicting the patient’s outcome; measure relative to the SVM chosen model (Entropy R2 is a FOness-of-fit measure for classification models. It represents the uncertainty in a dataset. Generalized R2 measures the proportion of variance in the dependent variable explained by the model. It is an extension of the traditional R-square used for linear regression and can be applied to non-linear models like SVM. Mean-log p (Mean Negative Log Likelihood) measures how well the predicted probabilities from the SVM model match the actual outcomes. Root Average Squared Error(RASE) measures the average squared difference between the predicted and actual values. Mean Absolute Deviation is the average of the absolute differences between the predicted and actual values). Line below: Variables in the SVM model and relative cost and gamma parameters; metrics and misclassification of the model if one of the variables is kept out. Considering the true positive (TP) and false positive (FP) classifications sensitivity (TP / (TP + FN)) and specificity (TN / (TN + FP)) indicate how well the model identifies positive and negatives cases respectively; Accuracy ((TP + TN) / (TP + TN + FP + FN)) measures the proportion of correctly classified cases among the total number of cases; balanced accuracy ((Sensitivity + Specificity) / 2) is the average of sensitivity and specificity; positive likelihood ratio (sensitivity / (1 - specificity)) represents how much more likely a positive result is to occur in people with the condition compared to those without it, while the negative likelihood ratio (1 - sensitivity) / specificity represent how much more likely a negative result is to occur in people without the condition compared to those with it; F1 score (2 * (precision * sensitivity) / (precision + sensitivity)) measures the model’s accuracy, combining both precision and recall into a single metric, where precision (TP / (TP + FP)) is the ratio between true positives and the sum of true positives and false positives.

In the HC vs. DoC classification, the best SVM model was obtained in the taVNS-R1 condition with a correct classification of HC and DoC of 87% and 84%, respectively, a misclassification rate of 11%, and a sensitivity, specificity, and accuracy of 80%, 90%, and 86%, respectively (Fig. 4). Of the 100 SVM-tested models, 55% gave a misclassification in training test below 20% and 33% below 15%.

**Fig. 5:**
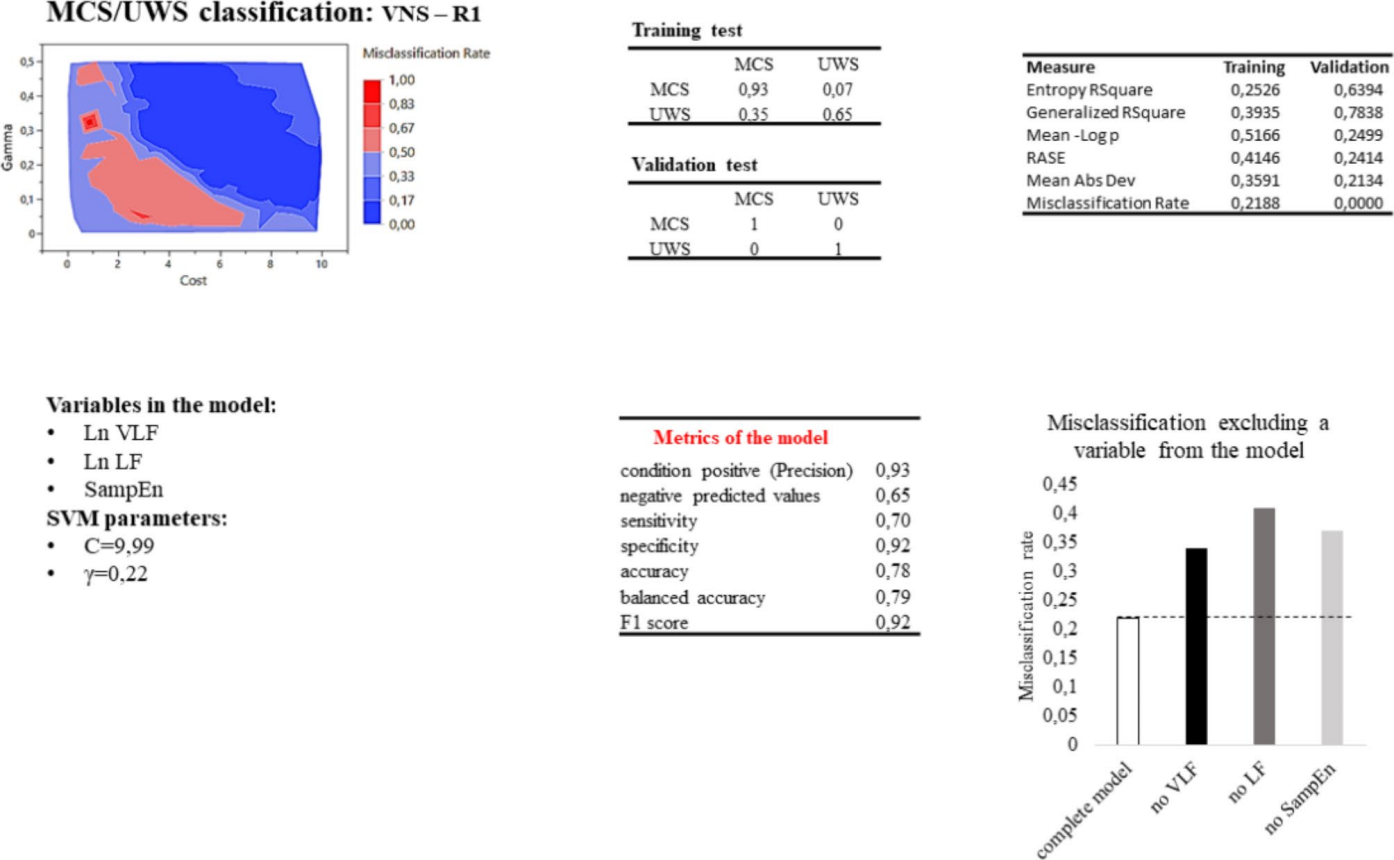
SVM MCS/UWS classification results. Line above: misclassification rate of 100 SVM models based on cost and gamma values; confusion matrix of the SVM model in predicting the patient’s outcome; measure relative to the SVM chosen model (Entropy R2 is a FOness-of-fit measure for classification models. It represents the uncertainty in a dataset. Generalized R2 measures the proportion of variance in the dependent variable explained by the model. It is an extension of the traditional R-square used for linear regression and can be applied to non-linear models like SVM. Mean-log p (Mean Negative Log Likelihood) measures how well the predicted probabilities from the SVM model match the actual outcomes. Root Average Squared Error(RASE) measures the average squared difference between the predicted and actual values. Mean Absolute Deviation is the average of the absolute differences between the predicted and actual values). Line below: Variables in the SVM model and relative cost and gamma parameters; metrics and misclassification of the model if one of the variables is kept out. Considering the true positive (TP) and false positive (FP) classifications sensitivity (TP / (TP + FN)) and specificity (TN / (TN + FP)) indicate how well the model identifies positive and negatives cases respectively; Accuracy ((TP + TN) / (TP + TN + FP + FN)) measures the proportion of correctly classified cases among the total number of cases; balanced accuracy ((Sensitivity + Specificity) / 2) is the average of sensitivity and specificity; positive likelihood ratio (sensitivity / (1 - specificity)) represents how much more likely a positive result is to occur in people with the condition compared to those without it, while the negative likelihood ratio (1 - sensitivity) / specificity represent how much more likely a negative result is to occur in people without the condition compared to those with it; F1 score (2 * (precision * sensitivity) / (precision + sensitivity)) measures the model’s accuracy, combining both precision and recall into a single metric, where precision (TP / (TP + FP)) is the ratio between true positives and the sum of true positives and false positives.

Similarly, in the MCS vs. UWS/VS classification, the best SVM model was obtained in the taVNS-R1 condition but with a lower performance. The model correctly classified 93% of MCS and 65% of UWS/VS, with a misclassification rate of 22% and a sensitivity, specificity, and accuracy of 70%, 92%, and 78%, respectively (Fig. 5). Of the 100 SVM-tested models, 33% gave a misclassification below 22% in the training test.

**Fig. 6:**
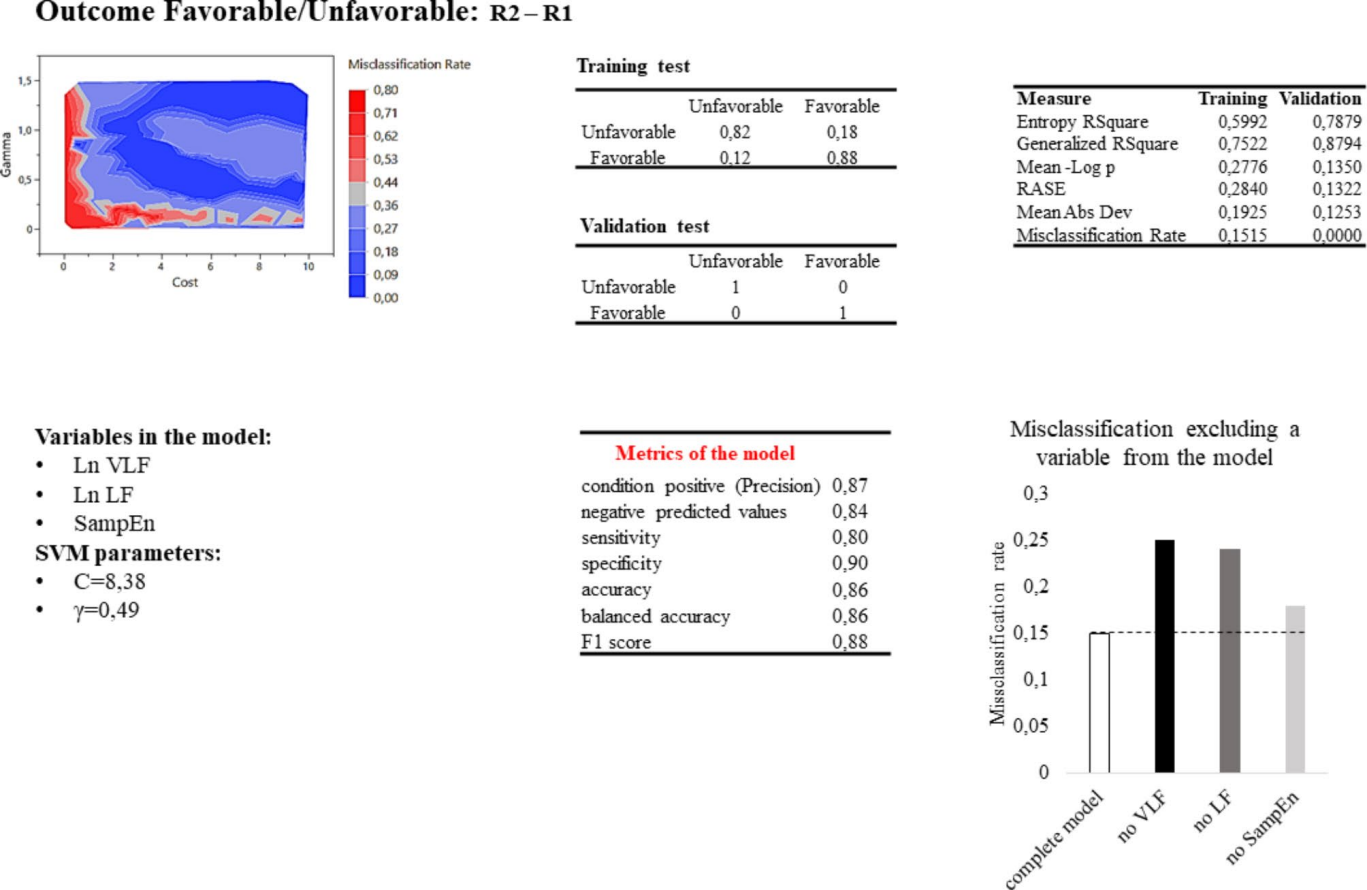
SVM Favorable/Unfavorable outcome prediction results. Line above: misclassification rate of 100 SVM models based on cost and gamma values; confusion matrix of the SVM model in predicting the patient’s outcome; measure relative to the SVM chosen model (Entropy R2 is a FOness-of-fit measure for classification models. It represents the uncertainty in a dataset. Generalized R2 measures the proportion of variance in the dependent variable explained by the model. It is an extension of the traditional R-square used for linear regression and can be applied to non-linear models like SVM. Mean-log p (Mean Negative Log Likelihood) measures how well the predicted probabilities from the SVM model match the actual outcomes. Root Average Squared Error(RASE) measures the average squared difference between the predicted and actual values. Mean Absolute Deviation is the average of the absolute differences between the predicted and actual values). Line below: Variables in the SVM model and relative cost and gamma parameters; metrics and misclassification of the model if one of the variables is kept out. Considering the true positive (TP) and false positive (FP) classifications sensitivity (TP / (TP + FN)) and specificity (TN / (TN + FP)) indicate how well the model identifies positive and negatives cases respectively; Accuracy ((TP + TN) / (TP + TN + FP + FN)) measures the proportion of correctly classified cases among the total number of cases; balanced accuracy ((Sensitivity + Specificity) / 2) is the average of sensitivity and specificity; positive likelihood ratio (sensitivity / (1 - specificity)) represents how much more likely a positive result is to occur in people with the condition compared to those without it, while the negative likelihood ratio (1 - sensitivity) / specificity represent how much more likely a negative result is to occur in people without the condition compared to those with it; F1 score (2 * (precision * sensitivity) / (precision + sensitivity)) measures the model’s accuracy, combining both precision and recall into a single metric, where precision (TP / (TP + FP)) is the ratio between true positives and the sum of true positives and false positives.

The best SVM model to predict the favorable vs. unfavorable outcome was obtained in the R2-R1 condition. The model correctly predicted 82% of unfavorable and 88% of favorable conditions, with a misclassification rate of 15% and a sensitivity, specificity, and accuracy of 80%, 90%, and 86%, respectively (Fig. 6). Of 100 SVM-tested models, 35% gave a misclassification below 20% and 7% below 15%.

To observe the influence of each variable, the SVM model was computed without the selected variable. In the SVM model to classify HC and DoC, the misclassification increased to 25%, excluding the VLF, 27% excluding the LF, and 32% excluding the SampEn from the model. In the same way, the misclassification of MCS and UWS/VS groups increased to 34%, excluding the VLF, 37% excluding the SampEn, and 41% excluding the LF from the model. Finally, in the Faforable/Unfavorable outcome, the misclassification increased to 18% excluding the SampEn, 24% excluding the LF, and 25% excluding the VLF from the model.

### Outcome at six month

After six months, of the 31 patients correctly predicted an outcome at three months, 9 (29%) changed diagnoses, with 5 with a pejorative trend (i.e., change from MCS to UWS).

## Discussion

This study sought to elucidate the influence of taVNS on HRV parameters in healthy subjects and patients with DoC, aiming to delineate outcome predictors. By examining HRV spectral components (VLF^35^, LF, and HF) and entropy (SampEn), we aimed to capture the sympathovagal dynamics and the complexity of brain-heart interactions in response to taVNS.

We observed significant changes in HF, VLF, and SampEn during the stimulation and recovery phases among HC and DoC patients. Notably, HC exhibited elevated HF during stimulation, while DoC patients showed higher VLF during recovery. Additionally, UWS/VS patients had increased VLF and SampEn during stimulation. However, the low effect size suggests the modest clinical significance of these differences.

The vagal system influences the HF band of the HRV, and its variation during the taVNS was documented in HC and DoC patients. A work of Geng and colleagues involving 44 healthy subjects and based on two different protocols (i.e., one to compare the effects of taVNS and sham taVNS on HRV and the other to test the effects of different taVNS parameters on the HF component of HRV)^36^, showed a significant increase of the vagal components during the taVNS, and that the sympathovagal balance, represented by the LF/HF ratio could significantly predict the response from participants to taVNS.

In their study on the impact of taVNS on the HR, Badran and his team^37^ set up the device with various parameters, observing that the HR dropped instantly as soon as the stimulation started (stimulation period) and remained at this lower level. When the stimulation ended (recovery period), there was an immediate surge in HR that went beyond the initial baseline for close to 30 s before it gradually returned to the average resting HR.

Similarly, Keute et al.‘s^38^ study on 44 HC confirmed parasympathetic activation following the taVNS but not after the stimulation.

In a case study of a patient with DoC, the long-term effects of taVNS on HR and HRV were studied^31^, showing that at the commencement of the taVNS program, the patient exhibited high pre-stimulation HR levels, indicative of a sympathetic nervous system predominance. Over time, a significant decrease in HR was documented, suggesting a shift towards parasympathetic dominance, corroborated by the trends in resting HRV-HF, reflecting long-term stabilization and enhancement of parasympathetic activity. These findings support the hypothesis that taVNS can lead to sustained changes in autonomic regulation. This study aligns with prior research by Clancy et al.^39^, further substantiating the impact of taVNS on autonomic balance.

Collectively, these studies provide a robust context for understanding the autonomic impacts of taVNS, affirming its role in modulating sympathovagal dynamics and underpinning the mechanistic rationale for the observed HRV changes in our study.

Although prior research on general taVNS impacts on HRV and vagal tone^40–42^, this study is novel in its examination of SampEn and VLF’s role—elements less documented in the literature but crucial for understanding long-term regulatory mechanisms like the renin-angiotensin-aldosterone system^43,44^.

An SVM approach was recently used to predict outcomes in 58 UWS/VS patients with high accuracy^45^, employing as predictors the time from injury, HF, SDNN (i.e., the cardiac variability) in resting state, and the SampEn during the stimulations.

In our study studying the impact of one session of taVNS, the SVM approach allowed us to differentiate between healthy subjects and DoC patients and within DoC subgroups. This approach achieved an 86% accuracy rate in predicting three-month outcomes, with a 71% confirmation at six months.

The ten-minute duration of each study phase was sufficient to observe taVNS’s modulation of HR entropy, sympathetic activity, and the VLF component, which is associated with long-term regulatory mechanisms like the renin-angiotensin-aldosterone system. These observations underscore the intricate responses within the CAN elicited by taVNS. As a reflection of CAN activity, the results provide insight into the potential effects of taVNS on this patient population, providing a possible outcome and suggesting that targeted modulation of these parameters could offer a novel avenue for enhancing autonomic regulation and possibly facilitating recovery.

In the field of consciousness recovery, particularly for patients with brain injuries and DoC, the role of taVNS remains a subject of ongoing research.

Researchers have suggested that taVNS can induce behavioral improvements in DoC patients at the clinical level with effects on neural activity at the neurophysiological level^30,31,46^.

Briand and colleagues^46^ proposed the Vagal Cortical Pathways model, based on the recovery mechanisms of consciousness and the *modus operandi* of taVNS, which outlines four sequential pathways: lower brainstem activation, upper brainstem activation, norepinephrine pathway, and serotonin pathway. Additionally, they suggest six mechanisms through which taVNS may influence brain activity in the process of consciousness recovery: activation of the ascending reticular activating system, thalamic activation, restoration of the cortico-striatal-thalamic-cortical loop, promotion of negative connectivity between the external and default mode networks via salience network activation, increased activity and connectivity in the external network through the norepinephrine pathway, and enhanced activity in the default mode network via the serotonin pathway.

A systematic review^47^ noted variable results in taVNS studies but confirmed patient improvements correlating with enhanced CRS-R scores and default mode network connectivity, highlighting the need for further research.

Our work innovatively proposes a method that combines HRV parameter analysis with taVNS to deeply explore identifiable differences in autonomic responses during taVNS in DoC patients with different levels of consciousness, especially for MCS and UWS/VS patients.We underscore the importance of examining brain-heart dynamics in DoC patients via taVNS. We leveraged HRV analysis as a key instrument to probe these interactions, utilizing a spectrum of HRV metrics to elucidate the autonomic effects of taVNS. Ensuring the integrity of our data, we maintained strict control over environmental variables such as temperature and lighting. Moreover, our adoption of SVM classification, validated through tenfold cross-validation, shows the promising role of machine learning in enhancing clinical predictions for DoC patients, supporting the model’s validity and potential for broader application, which is also expected to provide a new perspective and approach for clinical diagnosis and treatment.

### Limitations

Our study’s limitations need consideration. The relatively small sample size, despite rigorous statistical management, could potentially limit the results’ generalizability. Moreover, the uneven distribution of genders and the differences in time since injury and causes of conditions could have introduced biases. While ten-fold cross-validation lends credibility to our model’s reliability, further external validation across a broader and more diverse cohort is essential for substantiating our findings.

Nevertheless, our study provides significant insights into the utility of taVNS in evaluating DoC patients. The robust classification outcomes reflect that SVM modeling with an RBF kernel successfully navigates the trade-off between model complexity and generalization capability. Furthermore, the study is strengthened by the precise operational definition of ‘favorable’ and ‘unfavorable’ outcomes, grounded in observable clinical changes in consciousness, enhancing the practical application of our research. What is important to note is that due to the high misdiagnosis rate of DoC, the accuracy of the diagnosis is critical. According to the latest guidelines in the World Health Organization(WHO), recommended diagnostic methods include repeated clinical evaluation and at least one instrumental assessment, such as fMRI, PET, or EEG. These methods can provide insight into a patient’s brain function and help improve the accuracy of diagnosis. In this study, we present the assessment of the ANS as an innovative measure designed to complement existing diagnostic tools and provide additional information in multimodal assessment strategies. However, it should be seen as part of, and not as an alternative to, the currently proposed multimodal evaluation strategy.

## Data Availability

The data that support the findings of this study are available on request from the corresponding author upon reasonable request.
